# Decoding the research landscape of drug resistance and therapeutic approaches in head and neck cancer: a bibliometric analysis from 2000 to 2023

**DOI:** 10.3389/fphar.2024.1375110

**Published:** 2024-04-05

**Authors:** Qi Han, Junqi Shi, Jiaojiao Liu, Yang Fu, Zhongxun Li, Huina Guo, Xiaoya Guan, Xuting Xue, Hongliang Liu, Liting Zhao, Chunming Zhang

**Affiliations:** ^1^ Shanxi Key Laboratory of Otorhinolaryngology Head and Neck Cancer, First Hospital of Shanxi Medical University, Taiyuan, China; ^2^ Shanxi Province Clinical Medical Research Center for Precision Medicine of Head and Neck Cancer, First Hospital of Shanxi Medical University, Taiyuan, China; ^3^ Shanxi Key Laboratory of Otorhinolaryngology Head and Neck Cancer, The First Clinical Medical College of Shanxi Medical University, Taiyuan, China; ^4^ Department of Cardiology, Shanxi Cardiovascular Hospital, Taiyuan, China; ^5^ Department of Otolaryngology Head and Neck Surgery, First Hospital of Shanxi Medical University, Taiyuan, China; ^6^ Department of Cell Biology and Genetics, the Basic Medical School of Shanxi Medical University, Taiyuan, Shanxi, China

**Keywords:** head and neck cancer, drug resistance, bibliometric analysis, research frontiers, data visualization

## Abstract

**Introduction:**

Head and neck cancer is one of the most common tumors worldwide. However, drug resistance in its treatment has become a major factor limiting the efficacy. This study aims to comprehensively understand the current status of research in this field.

**Methods:**

The study analyzes papers related to therapeutic resistance in head and neck cancer published between 2000 and 2023 in the Web of Science Core Collection To achieve the research objectives, we searched the WoSCC for research and review papers on therapeutic resistance in head and neck cancer from 2000 to 2023, screened the English literature, and analyzed the research hotspots, academic collaborations, and trends in detail using tools such as Citespace, SCImago Graphica, and VOS viewer.

**Results:**

This study summarizes 787 head and neck cancer treatment resistance publications from WoSCC. The analysis showed that China and the United States are the major contributors in this field, and Grandis Jennifer R and Yang Jai-Sing are the key scholars. Keyword analysis showed that “cisplatin resistance” is a continuing focus of attention, while “Metastasis” and “Ferroptosis” may be emerging research hotspots. Literature clustering analysis pointed out that “Ferroptosis”, “Immunotherapy” and “ERK signaling” were the recent hotspots that received extensive attention and citations. Finally, we discuss the current status and challenges in drug-resistant therapies for head and neck cancer.

**Conclusion:**

This study is the first comprehensive bibliometric analysis of drug resistance in head and neck cancer. Reveals current trends and helps researchers grasp cutting-edge hotspots in the field.

## 1 Introduction

Head and neck cancer is a common malignant tumor worldwide. It covers a range of tumors in the oral cavity, nasal cavity, pharynx, larynx and neck (Tumban, 2019). The most common subtype of the disease is squamous cell carcinoma, while other types such as adenocarcinoma and small cell carcinoma are also included (Jumaniyazova et al., 2022). Drug-adjuvant therapies such as chemotherapy and immunotherapy play a key role in the treatment of head and neck cancer, improving surgical outcomes and reducing recurrence risk. (Harrington et al., 2023; Liu et al., 2024a). However, drug resistance during treatment limits long-term efficacy. In particular, drug resistance is driven by complex changes in cell signaling pathways (Jha et al., 2023; Trocchianesi et al., 2023), as well as the tumor microenvironment (Biswal et al., 2023; Qiao et al., 2023; Zhang et al., 2023). Therefore, an in-depth understanding and study of the molecular basis of drug resistance is essential for the development of more effective therapeutic strategies. Future studies should explore new drug combinations, targeted therapies, and individualized resistance prevention strategies. This will likely enable them to overcome drug resistance challenges in head and neck cancer treatment.

As technology advances, head and neck cancer treatment has seen an array of new drug treatment options, including some high-profile new drugs. First, anti-PD-1/PD-L1 immune checkpoint inhibitors, such as nivolumab (Marco et al., 2022) and pembrolizumab (Yuan et al., 2023), have made remarkable progress in head and neck cancer treatment in recent years (Bommireddy et al., 2020; Maroun and Mandal, 2021). These drugs have demonstrated excellent efficacy in some patients by activating the immune system and enhancing the body’s anti-tumor defense response. However, different individuals have different immune statuses, leading to variable therapeutic effects of these drugs. Secondly, targeted therapy has also become a major direction in head and neck cancer treatment (Stabile et al., 2013). For example, epidermal growth factor receptor (EGFR) inhibitors, such as cetuximab, inhibit tumor cell growth and division by interfering with the EGFR signaling pathway (Ratushny et al., 2009). Although these drugs have shown high efficacy in some patients, they are susceptible to drug resistance in long-term treatment, limiting their clinical application (Cserepes et al., 2022; Chan et al., 2023; Doghish et al., 2023). Finally, some herbal ingredients, such as baicalein in Scutellaria baicalensis, have antitumor activity (Tang and Dong, 2023). It has potential efficacy against drug-resistant head and neck cancer by inhibiting cancer cell proliferation and promoting apoptosis (Guo et al., 2019; Gao et al., 2020). In addition, artemisinin (Li, 2021) and its derivatives are complementary in traditional Chinese medicine (Roh et al., 2017). Studies have shown that they can regulate tumor cell signaling through multiple pathways and inhibit drug resistance in head and neck cancer. In general, head and neck cancer is treated with a variety of drugs, including immune checkpoint inhibitors, targeted therapies, and herbal treatments. However, differences in individual immune status and drug resistance due to long-term use are the main challenges to unstable therapeutic effects. Therefore, an in-depth study of drug resistance mechanisms in head and neck cancer is essential to optimize therapeutic strategies, overcome drug resistance, and promote innovative drug treatment options.

Bibliometrics is a method of assessing academic literature using mathematical and statistical methods (Wu et al., 2022). This approach allows us to analyze trends in a given field comprehensively. It also allows us to explore the contributions made by individuals from different institutions and countries. Medical fields have widely used bibliometrics as a research method in recent years (Cheng et al., 2022; Han et al., 2023). This paper is the first study to systematically analyze therapeutic drug resistance in head and neck cancer using bibliometric methods. We comprehensively applied bibliometric methods to analyze in-depth research advances in drug resistance in head and neck cancer, covering January 2000 to December 2023. This study is organized along six dimensions: 1) annual publication output and citation trends; 2) collaborative networks between countries and institutions; 3) author collaborative networks and contributions; 4) keyword co-occurrence and cluster analysis; 5) citation and cluster analysis of the literature; and 6) journal impact analysis. Combining the results of these analyses, we explore the current status and future direction of drug resistance research in head and neck cancer. At the same time, we also discuss the applications and challenges in this field. We aim to provide future researchers with novel perspectives and methods for drug resistance research in head and neck cancer.

## 2 Materials and methods

### 2.1 Data collection

The Web of Science Core Collection (WoSCC) is a comprehensive scholarly treasure trove covering more than 190 subject areas worldwide. The database is widely recognized as an outstanding resource for bibliometric research in a variety of disciplines, providing superior literature retrieval and citation analysis services (Han et al., 2023).

### 2.2 Bibliometric key terms

#### 2.2.1 Analysis of influential authors

H-index is a measure of scholarly output and impact, indicating that H papers published by an author have been cited at least H times (Hirsch, 2005; Harzing, 2016). G-index emphasizes papers with more evenly distributed citations on the basis of H-index (Egghe, 2006), and M-index is an improvement of H-index by considering the number of years of academic experience (von Bohlen Und Halbach, 2011). The combination of publication volume, H-index, G-index and M-index can be used to more comprehensively evaluate scholars’ academic contribution and influence.

#### 2.2.2 keywords and literature cluster analysis

In this study of keyword and literature co-citation clustering, the silhouette parameter is used to assess literature clustering quality (Rousseeuw, 1987). It has a value between −1 and 1, with closer to one indicating better clustering and closer to −1 indicating worse clustering. This parameter helps the researcher determine the separation and closeness of the literature points in the clusters.

In addition, LSI (Latent Semantic Indexing) and LLR (Log-Likelihood Ratio) are both algorithms applied for literature clustering. LSI uses Singular Value Decomposition (SVD) for dimensionality reduction to reduce the number of features and through semantic relatedness to map the literature in a low-dimensional space (Dumais, 2004). LLR uses a log-likelihood ratio test to find groups of significantly related literature by comparing lexical distributions among the literature (Glover and Dixon, 2004). Finally, the combined LSI and LLR algorithms for literature clustering analysis consider semantic and lexical relatedness. They improve a deeper understanding of literature sets’ multilevel structure.

### 2.3 Bibliometric visualization software

The study used bibliometric analysis and visualization software, including R 4.3.0, BiblioMetrix, Citespace, VOSviewer, SCImago Graphica, and the online tool MapEquation.

#### 2.3.1 R 4.3.0

Based on R version 4.3.0, we conducted a comprehensive analysis of the literature related to drug resistance in head and neck cancer through the Bibliometric package (Aria and Cuccurullo, 2017). First, by extracting and analyzing the annual keyword frequency and cumulative frequency, we revealed the hot areas of drug resistance research in head and neck cancer. Second, scholars with significant influence in this subject area were identified through detailed analysis of author contributions. Finally, we evaluated the impact of journals using Bradford’s Law to identify journals with significant status in head and neck cancer drug resistance research (Yatsko, 2012). This comprehensive scholarly analysis provides insights into key topics, journal quality, and scholarly contributions to head and neck cancer drug resistance research. This provides valuable research insights for the academic community.

#### 2.3.2 Citespace advanced (v6.2.R2 and v6.2.R4)

Citespace is software for visualization and econometric analysis of scientific literature. It is mainly used to reveal key themes, author collaboration networks and research dynamics in the academic field (Chen et al., 2010). This study reveals academic structure and research hotspots in the field by constructing author collaboration networks, keyword co-occurrence analysis and citation clustering. It can help researchers track author collaborations, discover research frontiers, and visualize literature citation networks. This helps to gain a deeper understanding of the whole picture of drug resistance mechanism research in head and neck cancer, and provides insights into the development of more effective therapeutic strategies.

#### 2.3.3 VOSviewer and SCImago graphica

VOSviewer software plays a key role in head and neck cancer drug resistance bibliometric analysis in synergy with SCImago Graphica software. With VOSviewer, researchers can conduct collaborative network analyses of countries and institutions (Perianes-Rodriguez et al., 2016; Measuring Scholarly Impact: Methods and Practice | SpringerLink, 2024). This will reveal patterns of collaboration among different countries and institutions involved in head and neck cancer drug resistance research. In addition, VOSviewer combined with SCImago Graphica’s visual clustering analysis enables researchers to gain a deeper understanding of the organizational structure, research themes, and institutional groups in the research field. This helps to identify potential research trends, strengthen international collaborations, and provide comprehensive collaborative insights into scientific research in the field of drug resistance in head and neck cancer. The combination of the two provides researchers with a powerful tool to facilitate international collaborations and advance the research field.

#### 2.3.4 MapEquation

By exporting the head and neck cancer drug resistance literature through Citespace and combining it with the online tool MapEquation (https://www.mapequation.org/apps/AlluvialGenerator.html), we present five consistently influential papers in a shock flow diagram (Edler et al., 2017). This comprehensive analysis covers the core literature in the citation network and reveals its continued impact on the field’s development. MapEquation efficiently displays the citation flow of the literature in an impact flow diagram. This highlights the citation relationships between the literature and its contribution to the academic field. This approach provides a clear and comprehensive visualization for a deeper understanding of the literature. It has sustained impact in the field of drug resistance in head and neck cancer, providing valuable visual insights for academic research.

### 2.4 Statistical data analysis

This study used R (v4.3.0) to perform descriptive statistics and visualization. The fitted model for the trend analysis of the number of articles is the formula = *y* ∼ poly (x, 2). The fitted curve is a quadratic polynomial model based on the year x) and the number of publications y). The trend of the number of publications and the yearly citation trend are visualized in this study using the ggplot2 package.

## 3 Results

**FIGURE 1:**
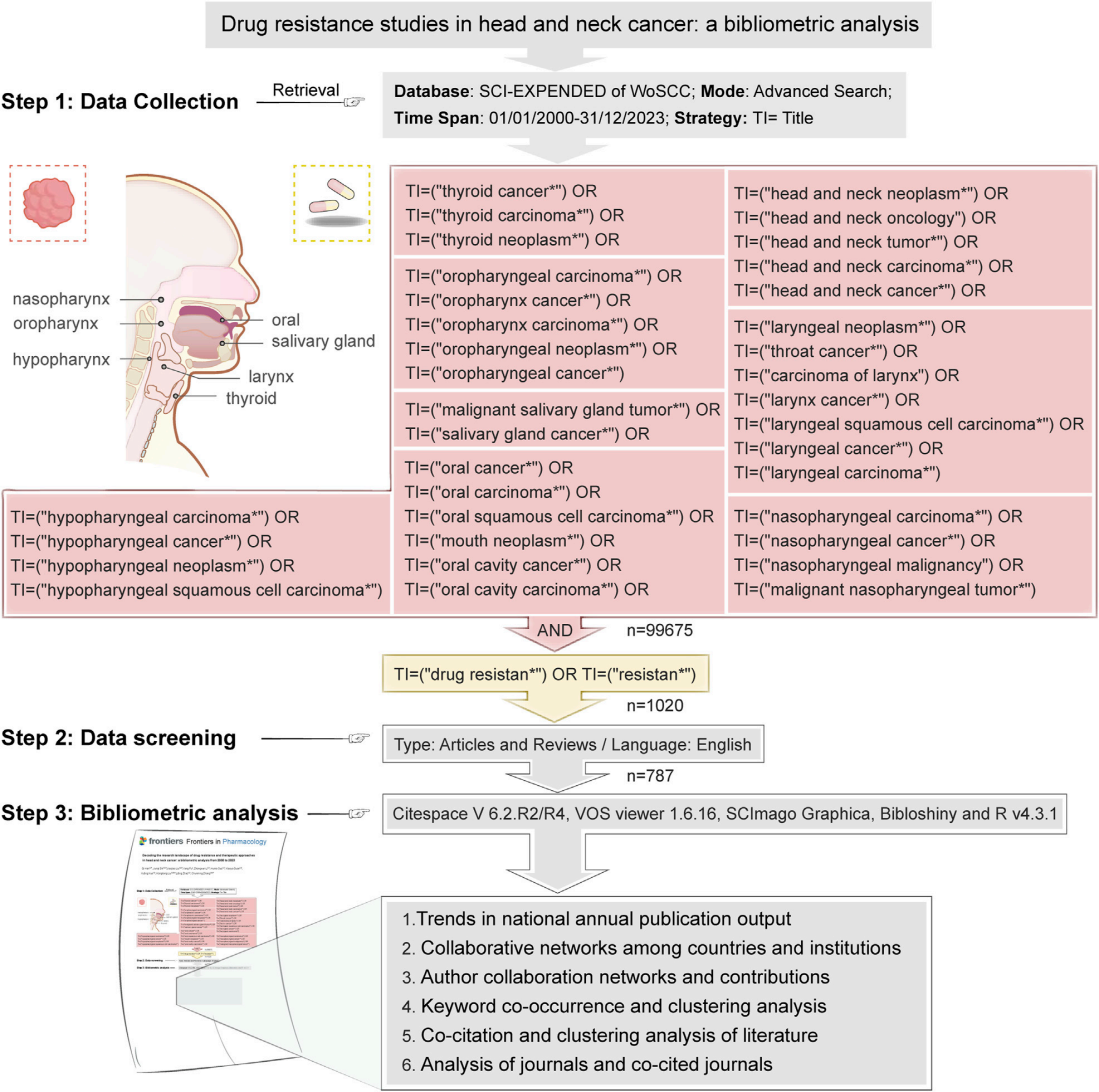
Literature strategy and data analysis flowchart.

Figure 1 describes our elaborate retrieval method. By performing a title search (TI) in the WoSCC database, we successfully obtained 1020 original articles. Subsequently, 786 articles were finalized by filtering them according to year of publication (2000.01-2023.12), type of publication (articles and reviews), and language (English). We then exported these retrieved documents in plain text form and used the downloaded bibliographic records for bibliometric analysis.

### 3.1 Annual publication and citation trends

**FIGURE 2:**
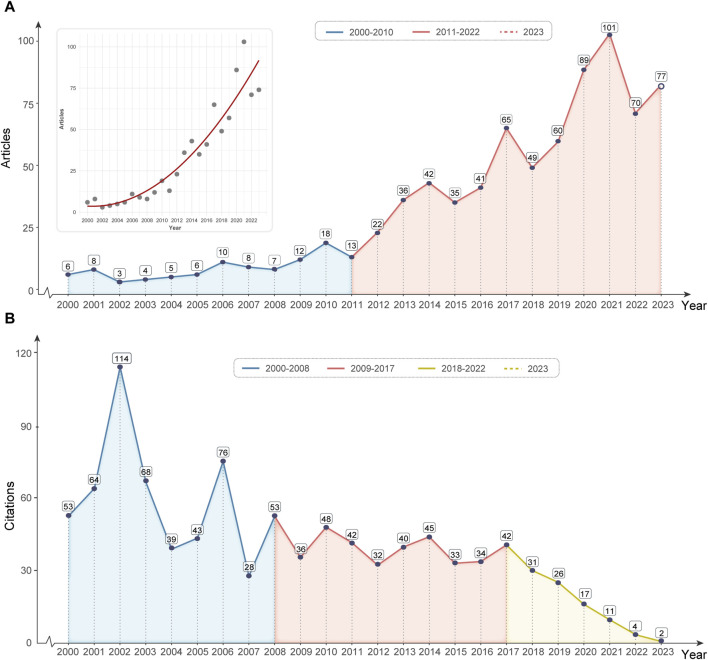
Annual publication and citation trends. **(A)** The upper left panel shows the pattern of annual publication growth using a non-linear fitting method. Between 2000 and 2010, the blue area corresponds to a trend of flat growth in annual publications. Between 2011 and 2023, the red area reflects a markedly rapid increase in annual publications. **(B)** The evolution of the annual citation trend can be divided into three phases. The blue interval shows fluctuations, the red interval characterizes a period of stabilization, and the yellow interval marks a phase of declining citations.

We used line graphs to present in detail the trend of growth in the number of papers and the trend of annual citation frequency in the field of head and neck cancer drug resistance research in the WoSCC database for the period from 2000 to 2023. In the overall study, we used R to fit a nonlinear model. This reveals the macro-trend that academic publications in this field are on the rise. In addition, we analyze this trend in depth by phase. Figure 2A shows a relatively flat growth trend in paper numbers between 2000 and 2010. Between 2011 and 2022, however, the number of papers in this field increased significantly. Taken together, our analysis reveals that drug resistance research in head and neck cancer has flourished, especially in recent years. This provides valuable insights into academic medicine.

In addition, we analyzed annual citation trends. As shown in Figure 2B, the trend can be divided into three parts: fluctuating (2000-2008), stable (2009-2017), and decreasing year by year (2018-2023). Combined with the trend of publications, we come up with five reasons: 1) Low-quality research: the increased number of publications may originate from low-quality or insufficiently influential research, which fails to attract other scholars’ attention and citations. Fragmentation of the field: As the number of publications increases, research may become more fragmented rather than concentrated on a few high-impact papers, which may lead to a decline in overall citations. 3) Citation lag: It may take time for citations to reflect the impact of research, and if the latest literature has not yet been fully cited, a decline in citations may be observed initially. 4) Changes in research direction: Changes in research direction within the field may cause a decline in citations. Citations may decrease if the proposed direction is not widely recognized or does not attract enough interest from the academic community.

In summary, head and neck cancer drug resistance research has shown significant growth between 2000 and 2023, especially explosive growth in recent years. The annual citation trend undergoes three phases of fluctuation, stabilization, and gradual decrease. This may be influenced by factors such as low-quality studies, field fragmentation, citation lag, changes in research direction, and potential data insufficiency or short-term fluctuations. These findings provide deep insights into the current state and trends of the field domain.

### 3.2 Analysis of national publications and institutional cooperation

**FIGURE 3:**
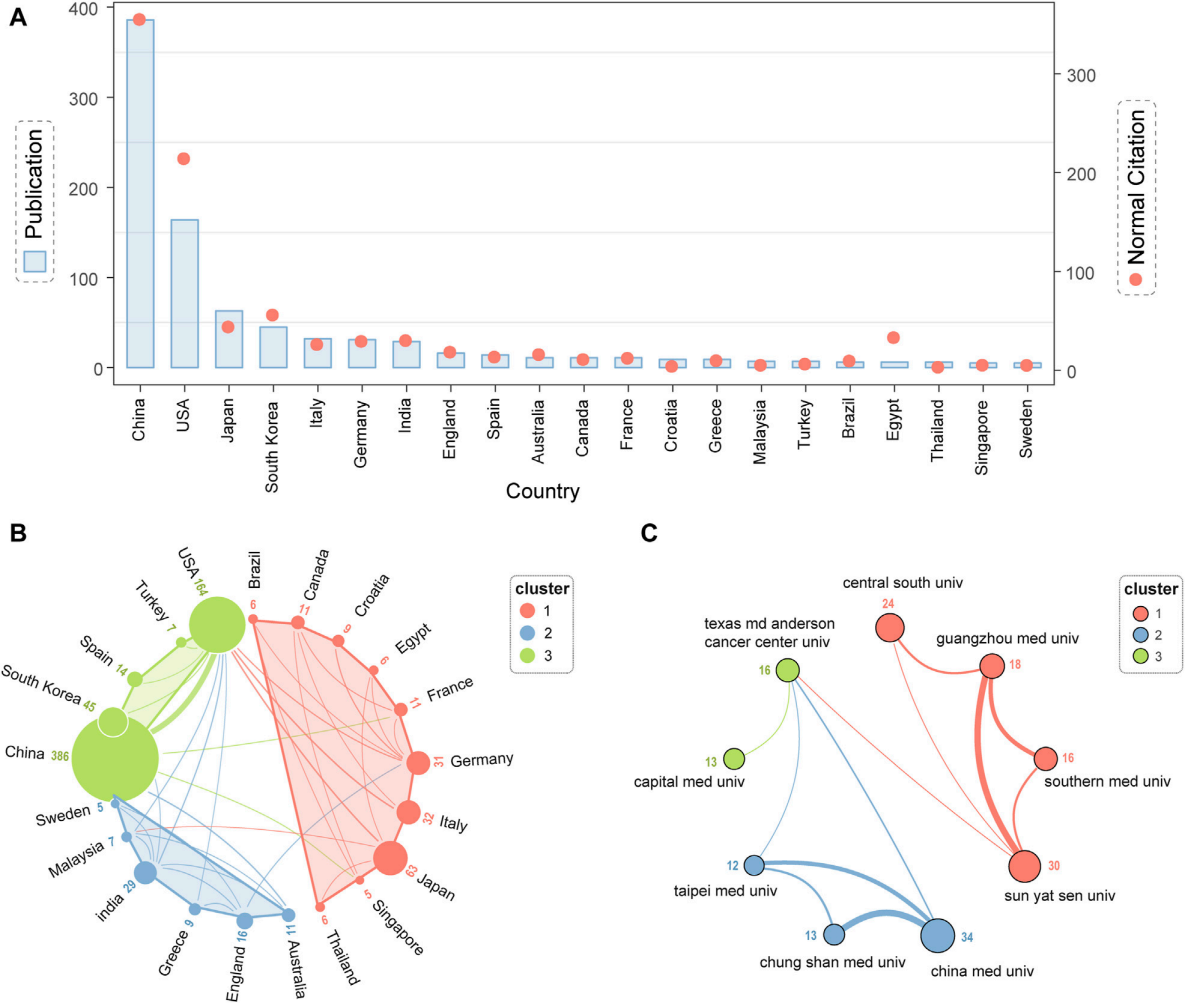
National, institutional and author contributions. **(A)** In the figure, the left *Y*-axis shows the number of publications, the right *Y*-axis shows citation frequency, and the *X*-axis shows countries. The blue histogram indicates that China has the most publications on the *X*-axis. The red dots represent the standardized citation frequency, which decreases from left to right. **(B)** Collaboration between countries is categorised in the figure by red, blue, and green. Dot sizes reflect the number of publications by country, while connecting lines reflect cooperation intensity. **(C)** The figure categorizes inter-agency collaborations in red, blue, and green. Size of dots indicates volume of publications, whereas the width of connecting lines indicates intensity of cooperation.

The analysis of national publications and citation frequency provides a powerful tool for understanding the scientific ecosystem, international partnerships, and assessing a country’s position and influence in the field of head and neck cancer drug resistance research. Supplementary Table S1 analyzes national publication volume and citation frequency. Figure 3A shows that China, the United States, and Japan are in the top three countries in terms of national publications. In addition, China, the United States, and South Korea are in the top three countries in terms of standardized citations. These figures may reflect the overall research strength and activity of China, the U.S., and Japan in this field. In addition, the citation performance hints at these countries’ academic influence. South Korea’s performance in citation frequency compared with Japan may imply that its research in the field or direction of head and neck cancer drug resistance research has relatively higher academic value.

In addition, we analysed the collaborative connections between countries and institutions in drug resistance research in head and neck cancer. This was done to reveal the current status of international academic exchanges in this field. First, in Figure 3B, the strength of collaborative connections between countries is categorized into three clusters, among which the strength of collaboration between China and the United States is high and in the same category. Second, the United States acts as a key cooperation bridge between these three clusters. In addition, Figure 3C shows that Chinese medical universities lead the number of publications in this research area. Finally, Texas M. D. Anderson Cancer Research Center, on the other hand, acts as a link between these three clusters. It provides key support for drug resistance research in head and neck cancer.

### 3.3 Author contributions and cluster analysis

In the field of drug resistance research in head and neck cancer, an in-depth analysis of the authors’ contributions is intended to provide a better understanding of the dynamics of scientific research in the field, especially those researchers who have achieved remarkable results in overcoming therapeutic resistance.

**FIGURE 4:**
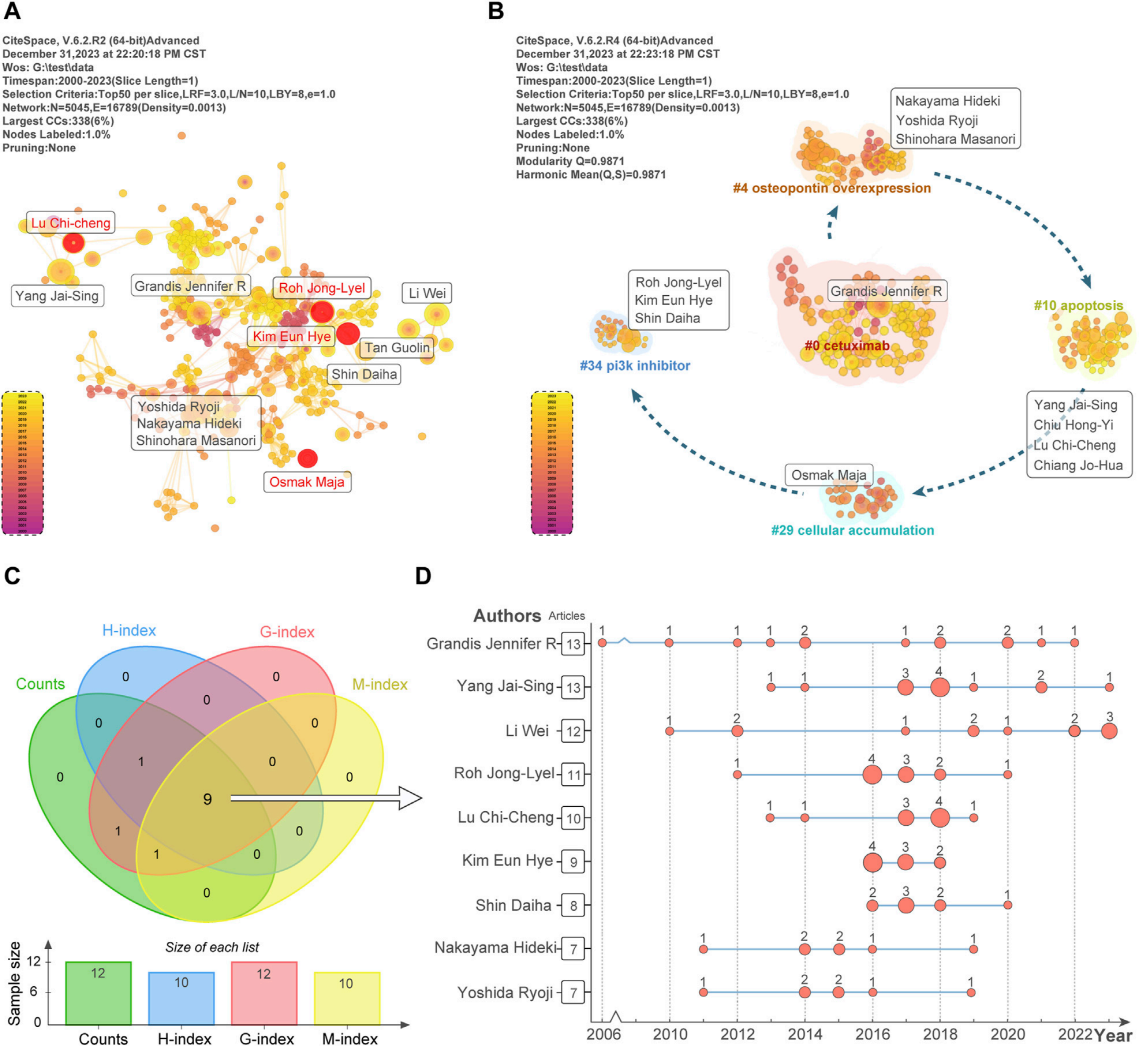
Author contributions and cluster analysis. **(A)** TOP10 publications and TOP4 burst strength author visualizations. **(B)** Cluster analysis of TOP10 published authors yielded five clusters representing their research areas. **(C)** Nine significant scholars were identified through a Venn diagram analysis of TOP10 publication volume, H-index, G-index, and M-index. **(D)** The vertical coordinates of the graph indicate the annual number of articles and total publications by nine influential scholars. The horizontal coordinates indicate the annual number of articles. The size of the red dots reflects each scholar’s publications.

Figure 4A visualizes the top 10 authors in terms of posts and highlights Citespace software users. Among the top 10 authors in terms of posts, Grandis Jennifer R and Yang Jai-Sing are at the top of the list with 13 posts each. In addition, the authors highlighted in red, Osmak Maja, Kim Eun Hye, Roh Jong-Lyel, and Lu Chi-Cheng, excelled in citations and were the most popular authors in the TOP4. Meanwhile, in Supplementary Figure S1, we present a timeline plot of the citation prominence of these four authors. Among them, Kim Eun Hye has the highest citation burst intensity of 4.32, lasting from 2016 to 2018.

In order to gain a deeper understanding of the research direction of authors in the field of head and neck cancer drug resistance research, we used LLR cluster analysis to obtain 46 clusters, in which the clusters from #0 to #46 were arranged in decreasing order of the number of authors. Meanwhile, analyzing the authors in combination with the TOP10 authors, we found that these authors were covered in 5 clusters. In Figure 4B, we show that authors with TOP10 publication volume were clustered into “#0 cetuximab”, “#4 osteopontin overexpression”, “#10 apoptosis”, “#29 cellular accumulation”, and “#34 pi3k inhibitor” in these five taxa. Therefore, through the analysis, we learned that these five taxa represent the research directions of TOP10 posting authors in the field of head and neck cancer drug resistance research.

In addition, we counted the publication volume, H-index, G-index and M-index of the TOP10 authors in this field in Supplementary Tables S2–S5, and further evaluated their scholarly influence and contribution to the field of head and neck cancer drug resistance research. First, in Figure 4C, we take the intersection of the authors of the TOP 10 of the above four indices using a Wayne diagram. This will enable us to obtain nine authors. Second, Figure 4D shows the annual publication volume of these nine authors. These nine authors have significantly contributed to the progress and development of head and neck cancer drug resistance research. They have done this with their excellent research output, high academic impact, and wide number of citations. Finally, Table 1 shows the institutions, publication volume, H-index, G-index, M-index, and total citation frequency of these nine authors. Their research results are of significant significance in guiding academic development and clinical practice of drug resistance research in head and neck cancer.

**TABLE 1:** The nine influential authors: Institution, counts, H/G/M index, and total citations.

Rank	Authors	Institution	Counts	H_index	G_index	M_index	TC
1	Yang Jai-Sing	China Medical University (Taichung)	13	12	13	1.091	583
2	Grandis Jennifer R	University of California	13	10	13	0.556	731
3	Lu Chi-Cheng	National Taiwan University of Sport	10	10	10	0.909	490
4	Roh Jong-Lyel	CHA University School of Medicine	11	10	11	0.833	1218
5	Kim Eun Hye	University of Ulsan College of Medicine	9	9	9	1.125	1159
6	Li Wei	Weill Cornell Medicine	12	8	12	0.571	168
7	Shin Daiha	Korea Basic Science Institute	8	8	8	1	1140
8	Nakayama Hideki	Kumamoto University	7	7	7	0.538	250
9	Yoshida Ryoji	Kumamoto University	7	7	7	0.538	250

### 3.4 Keyword co-occurrence and cluster analysis

**FIGURE 5:**
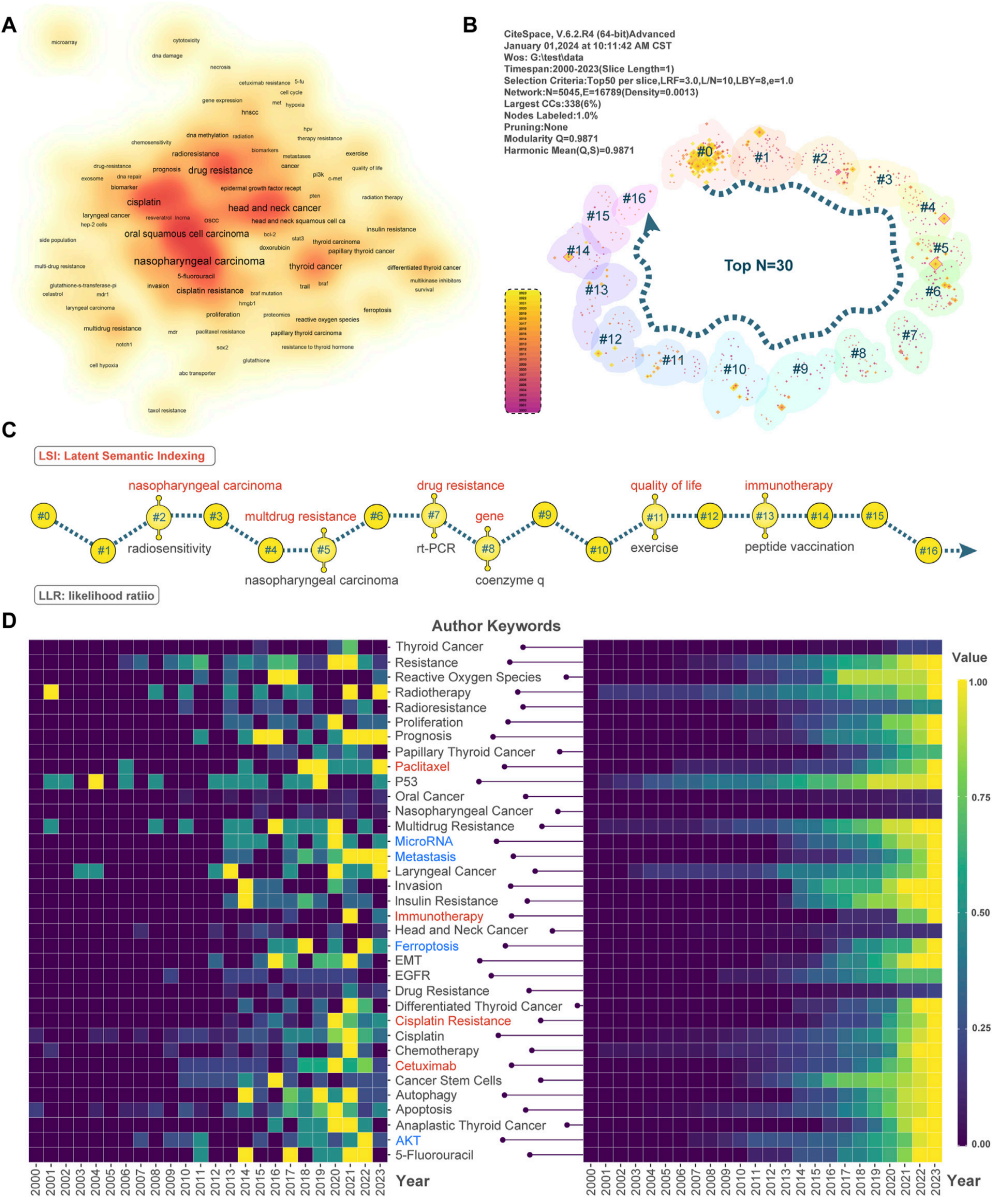
Keyword hotspots and cluster analysis. **(A)** The VOSviewer displays resistance keywords to head and neck cancer. Darker shades indicate a higher frequency of keywords. **(B)** By clustering the keywords based on N = 30, 16 clusters were obtained. As the arrows point down, the number of keywords in the clusters decreases. **(C)** Sixteen clusters were unfolded and labels were added using the LSI and LLR algorithms, respectively. **(D)** The left side of the panel shows keyword yearly frequency and the right side shows keyword cumulative frequency. While keyword frequencies have been normalized, the vertical axis represents keywords and the horizontal axis represents years.

Keyword co-occurrence analysis of drug resistance studies in head and neck cancer can reveal the interrelationships between keywords in head and neck cancer drug resistance studies, which helps to identify research hotspots and potential association mechanisms. We performed statistics and visualization of author keywords with VOSViewer. Figure 5A shows the keyword heat map, with darker colors representing higher keyword frequencies. In order to gain a deeper understanding of the hotspots and trends in drug resistance research in head and neck cancer, we categorized the keywords for frequency analysis. First, in Table 2, we categorized the keywords into 3 types of treatment methods, drug-resistant types, and research topics, and counted the frequency of TOP10 keywords in these three categories. In head and neck cancer drug-resistant treatment methods, cisplatin, cetuximab and paclitaxel keywords were Top 3. Cisplatin-resistant, anti-radiation and multi-resistant keywords were Top 3 in the drug-resistant group, and apoptosis, autophagy, EMT, EGFR and Cancer stem cell keywords were Top 3 in the research theme group. These high-frequency keywords reflect ongoing research hotspots. Secondly, we supplemented the statistics of keyword frequency in Supplementary Tables S6, S7 for therapeutic approach and research topic groups. Among them, drugs such as tetrandrine, temsirolimus, and triptolide in the drug therapeutics group potentially increase the sensitivity of anti-head and neck cancer drugs. And keywords such as extracellular vesicles, tumor microenvironment, and ferroptosis in the research topic group may reflect potential research hotspots in this field.

**TABLE 2:** Top 10 treatment approaches, drug resilience, and study themes keyword frequency.

Rank	Treatment approaches	Counts	Drug resilience	Counts	Study themes	Counts
1	cisplatin	63	cisplatin resistance	50	apoptosis	68
2	cetuximab	26	radiation resistance	38	autophagy	26
3	paclitaxel	19	multiple drug resistance	22	EMT	22
4	5-fluorouracil	14	insulin resistance	12	EGFR	22
5	immunotherapy	9	paclitaxel resistance	9	cancer stem cells	22
6	doxorubicin	8	cetuximab resistance	4	AKT	19
7	vemurafenib	8	resistance to thyroid hormone	4	metastasis	17
8	lenvatinib	7	erlotinib resistance	2	microRNA	16
9	sorafenib	7	immunotherapy resistance	2	p53	15
10	resveratrol	4	5-fu resistance	2	prognosis	14

Keyword clustering analysis, on the other hand, groups high-value keywords. This provides researchers with a systematic understanding of the drug resistance mechanism of head and neck cancer and a direction for in-depth exploration. We used Citespace software for LLR and LSI cluster analysis and keyword visualization. We set the clustering parameter to TOP N = 30/40/50, and obtained 16, 15, and 16 clusters, respectively (Figure 5B, Supplementary Figures S2, S3). Figure 5B shows that the TOP N = 30 visualization result contains 16 keyword clusters. The number of keywords in the clusters decreases in the direction of the arrows. In addition, we analyze the same clusters using different clustering algorithms. This is to determine the research direction of the #2, #5, #7, #8, #11, and #13 clusters. LSI clustering is shown in red font and LLR clustering is shown in black font in Figure 5C. #2 and #5 demonstrate multi-resistance *versus* radiotherapy sensitivity in nasopharyngeal cancer. #7, on the other hand, indicates that RT-PCR is a commonly used tool in drug resistance studies. #8 shows that coenzyme Q-related genes are associated with drug resistance. #11 reflects the relationship between recovery training and survival quality. Finally, #13 reflects the potential value of peptide vaccines as immunotherapeutic tools in anti-head and neck cancer resistance studies.

Further, we are interested in revealing the current research focus, research trends and innovative directions in drug resistance in head and neck cancer. We will do this by visualizing the annual keyword frequency heatmap based on keyword set theory and topic network construction. Figure 5D heatmap presents the normalized frequency of authors’ keywords during 2000–2023, with annual keyword frequency on the left and cumulative annual keyword frequency on the right. In terms of drug therapy, paclitaxel, immunotherapy, cisplatin resistance and cetuximab constitute the focus of drug resistance research in head and neck cancer. In terms of thematic research, “MicroRNA”, “Metastasis”, The keywords “Ferroptosis” and “AKT”, highlighted in blue, have become an emerging focus in the field of drug resistance research in head and neck cancer. In addition, in the upper right quadrant of Supplementary Figure S4, “cetuximab resistance”, “cisplatin-resistance”, “erlotinib ", “cisplatin-resistance”, “erlotinib” and “cell hypoxia” belong to the motor themes. These keywords have strong centrality and high density in head and neck cancer resistance research. They are conceptually closely related to other quadrant themes. The keyword categories “microarray” and “MTT assay” in the upper left quadrant have well-defined internal connections, but external connections are not relevant. The keywords in the lower left quadrant have themes that are neither developed nor significant. Topics in the lower right quadrant have relevant keywords to the research area, but are not developed. In summary, we have provided a comprehensive description of the hotspots and trends in drug resistance research in head and neck cancer. This is done through co-occurrence and clustering analysis of keywords.

### 3.5 Literature co-citation and cluster analysis

**FIGURE 6:**
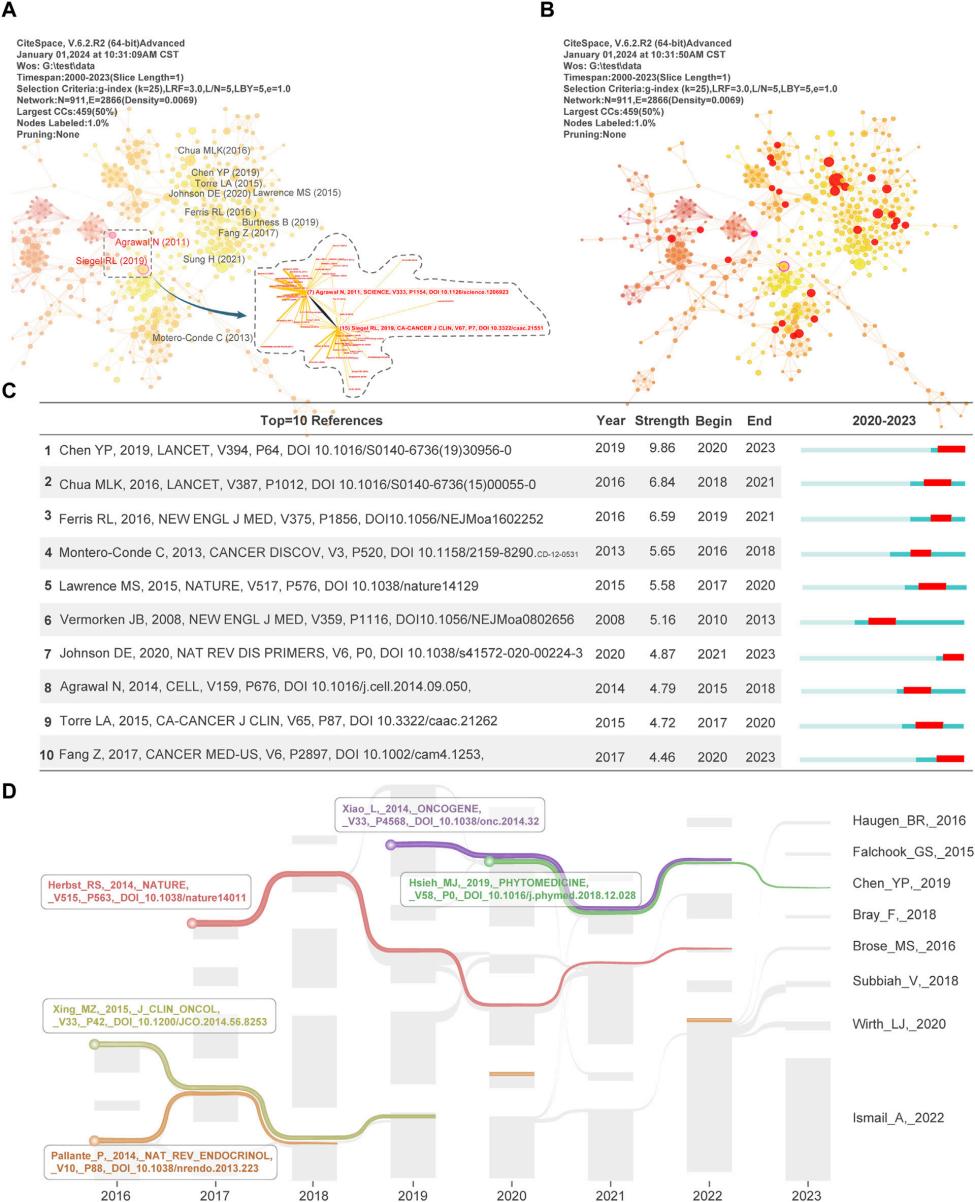
An analysis of the literature’s impact on the field. **(A)** Two papers with high performance in mediation, Agrawal et al. (2011) and Siegel et al. (2019), are shown in the figure, and they are presented visually with the top10 papers in terms of citation frequency. **(B)** The red dots in the graph indicate papers in the network with high burst intensity. **(C)** The panel shows the top10 papers in terms of outbreak intensity and duration. **(D)** By applying MapEquation to literature with citations that lasted more than 4 years or more, we successfully visualized five articles between 2016 and 2023.

Our literature co-citation and clustering analyses provide insights to systematically understand the hotspots of drug resistance research in head and neck cancer, assess the impact of scientific research, and reveal correlations between related subfields. Figure 6A presents the TOP10 most cited authors, with red highlighted authors. Siegel RL’s “*Cancer statistics, 2019*” (Siegel et al., 2019) and Agrawal N’s “*Exome sequencing of head and neck squamous cell carcinoma reveals inactivating mutations in NOTCH1*” (Agrawal et al., 2011) are the papers with the two authors with the high centrality. These two papers play a key role in bridging networks of information dissemination and communication. Academically, their contributions are highly influential and leading. Meanwhile, we present the papers that exploded in the field in Figure 6B using red circles based on the above. Further, we count the TOP 10 papers in terms of outbreak intensity in Figure 6C. During 2018–2023, the outbreak intensity of Chen YP (Chen et al., 2019) and Chua MLK (Chua et al., 2016) papers on Nasopharyngeal carcinoma was 9.86 and 6.84, respectively. This indicates that Nasopharyngeal carcinoma has been the focus of drug-resistant research in head and neck cancer for 5 years and is developing rapidly. In addition, we visualized the shock flow map of citation duration in Figure 6D. We screened the key papers that were consistently cited for more than 4 years. First, Xiao L and Hsieh MJ’s studies on improving cisplatin resistance and sensitivity to radiotherapy in nasopharyngeal carcinoma, respectively, received sustained attention (Xiao et al., 2014; Hsieh et al., 2019). Second, Herbst RS’s study on the effect of the PD-L1 antibody MPDL3280A on patients expressing high levels of PD-L1 in a variety of cancer types has been consistently cited for 6 years (Herbst et al., 2014). Finally, Xing MZ’s research on the mechanism of BRAF V600E mutation in thyroid cancer progression has been profoundly influenced by scholars in the field (Xing et al., 2015). In particular, the microRNA downregulation of Pallante P in thyroid cancer treatment and diagnosis continued to receive attention during 2016–2022 (Pallante et al., 2014). In summary, we dissected the key literature in drug resistance research in head and neck cancer from multiple perspectives. We provided insightful academic references for researchers, policymakers, and the academic community.

**FIGURE 7:**
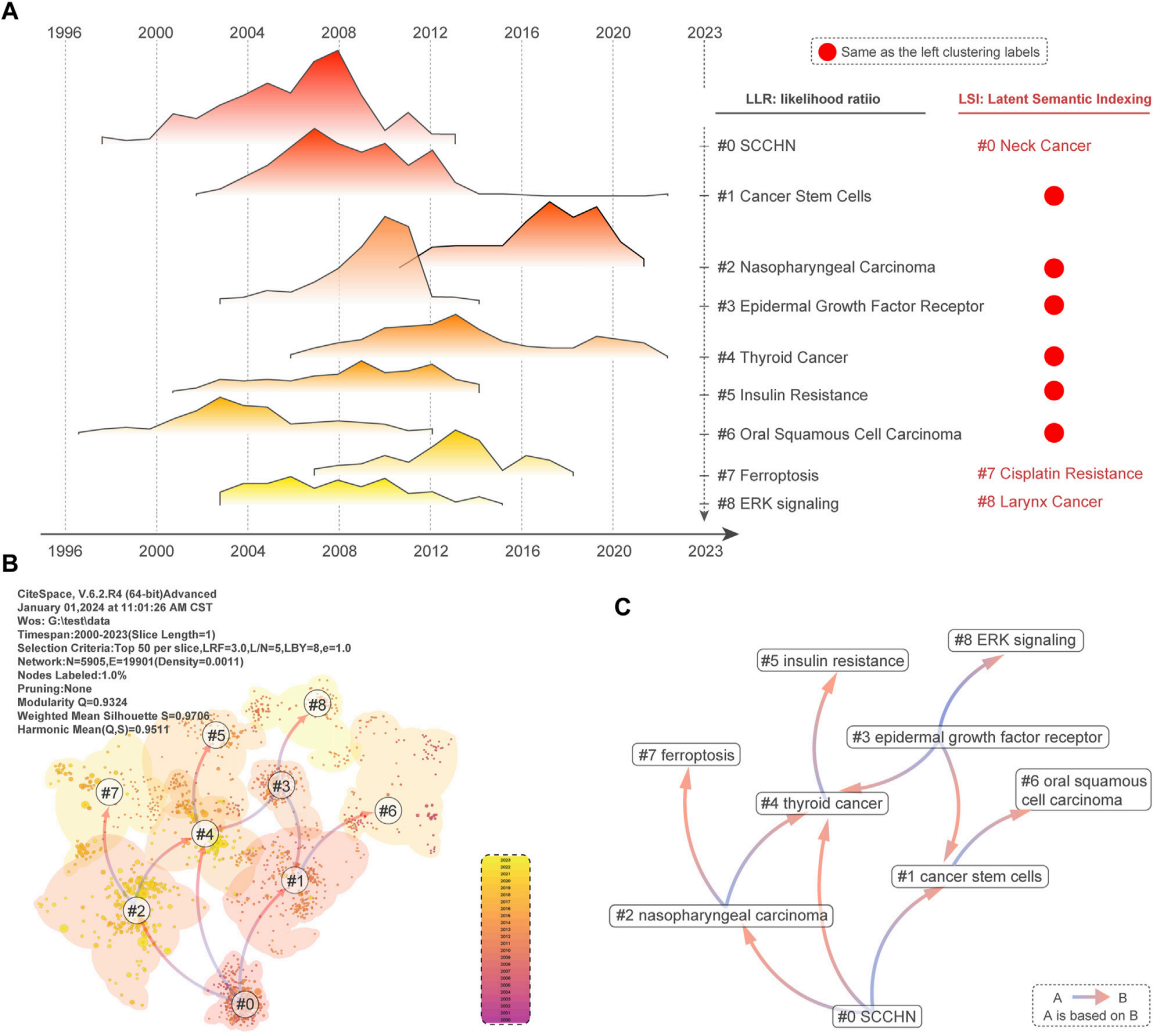
Clustering of literature based on visual analysis. **(A)** Literature clustering was analyzed and clusters 0#-#8 were selected to be visualized using a peaks and valleys graph. Labels obtained from LSI and LLR algorithms for clustering are vertical. Red points indicate that both algorithms agree on the labels. **(B)**The “Cluster Dependencies” function was used to visualize the dependencies between the nine clusters. References from A to B are indicated by arrows. **(C)** We abstracted the dependencies between clusters 0 to 8 and added the corresponding theme names.

In addition, clustering analysis by literature co-citation helps us dig deeper into the knowledge structure and research focus of drug resistance research in head and neck cancer. We obtained 9 clusters, #0-#8, after keyword and title clustering and filtering the literature by citespace. We labeled the clusters using LSI and LLR algorithms. The size, silhouette, Mean, LSI, and LLR information of each cluster in the results of keyword clustering of the literature is shown in detail in Table 3. Further, we deeply analyzed the hotspot and field variation of drug resistance research in head and neck cancer. We did this by the dependency between the taxon labeling and the previous taxon. First, in Figure 7A, the time-series changes in the number of publications of the nine taxa are presented, and the vertical coordinates are the LLR and LSI labels of different taxa. Among them, “#1 Cancer Stem Cells” is a cluster containing 257 publications, suggesting that cancer stem cells may be the focus of attention in drug resistance research in head and neck cancer. Secondly, taxa #2-#6 LLR and LSI classification labels were consistent and contained a decreasing number of literature based on the taxon number, reflecting the hot topic of drug resistance research in head and neck cancer. However, taxon #7 represents “Ferroptosis” and “Cispaltin Resistance” respectively, which may indicate that the relationship between iron death and cisplatin resistance mechanism has received attention from scholars. Similarly, taxon #8 may indicate the correlation between drug resistance and ERK pathway in laryngeal squamous carcinoma. Finally, we visualized the dependencies between different clusters in the keyword and title clustering of #0-#8 literature through the citespace “Cluster Dependencies” module in Figure 7B and Supplementary Figure S5A, respectively. By analyzing the dependencies among different clusters, we are able to deduce the evolutionary patterns of the themes of each cluster. Finally, for ease of analysis, we simplify this evolutionary pattern in Figure 7C. Clearly, "#1 Cancer stem cell”, "#2 nasopharyngeal carcinoma” and "#4 thyroid cancer " developed into the "#0 SCCHN” theme. Similarly, the dependency analysis presenting the clustering results of the literature titles in Supplementary Figure S5 showed that the "#0 targeting EGFR resistance network” theme was built on the “#1 stem-like cell” and “#4 thyroid cancer” themes. “ and ”#2 thyroid cancer” clusters.

**TABLE 3:** Literature cluster analysis: #0-#8 cluster sizes, silhouette values, means, and labels.

Cluster	Size	Silhouette	mean (Year)	Label (LSI)	Label (LLR)
0	333	0.834	2006	neck cancer; drug resistance; gene copy number; treatment response; predictive marker | stat; pi3k; grb2; pten; plc-gamma	scchn (12.27, 0.001); pten (6.91, 0.01); egfrviii (6.11, 0.05); predictive marker (6.11, 0.05); gene copy number (6.11, 0.05)
1	257	0.962	2008	oral squamous cell carcinoma; cancer stem cells; side population; sphere formation; to-mesenchymal transition | side population; drug resistance; sphere formation; to-mesenchymal transition; epidermal growth factor receptor	cancer stem cells (21.01, 1.0E-4); oral squamous cell carcinoma (9.11, 0.005); side population (6.51, 0.05); invasion (6.51, 0.05); carbon ion irradiation (6.51, 0.05)
2	199	0.947	2016	nasopharyngeal carcinoma; cisplatin resistance; fusion genes; microsatellite instability; mir-619-5p | oral squamous cell carcinoma; fusion genes; microsatellite instability; mir-619-5p; cell growth	thyroid cancer (10.04, 0.005); nasopharyngeal carcinoma (9.45, 0.005); cetuximab (6.78, 0.01); tumor microenvironment (6.46, 0.05); extracellular vesicles (6.46, 0.05)
3	188	0.956	2009	epidermal growth factor receptor; oral squamous cell carcinoma; sphere formation; cancer stem cells; stat | pi3k; grb2; pten; plc-gamma; c-met	epidermal growth factor receptor (5.82, 0.05); head and neck (4.75, 0.05); receptor tyrosine kinase (4.75, 0.05); review (4.75, 0.05); antibody (4.75, 0.05)
4	185	0.947	2013	thyroid cancer; braf mutation; braf inhibitors; radioactive iodine resistance; radioiodine refractoriness | drug resistance; signal transduction; molecular mechanisms; sodium iodide symporter; mouse model	thyroid cancer (38.77, 1.0E-4); vemurafenib (12.2, 0.001); kinase inhibitors (12.2, 0.001); drug resistance (11.52, 0.001); radioactive iodine refractory (8.12, 0.005)
5	136	0.977	2012	neck cancer; paclitaxel resistance; targeted therapy; immune infiltration; pi3 kinase class iii | cisplatin resistance; glutamate antiporter; lipid reactive oxygen species; pi3 kinase class iii; epigallocatechin gallate	ferroptosis (17.71, 1.0E-4); reactive oxygen species (13.68, 0.001); nrf2 (9.1, 0.005); resveratrol (9.1, 0.005); human cisplatin-resistant oral cancer car cells (9.1, 0.005)
6	128	0.997	2008	insulin resistance; igfbp-3 gene polymorphisms; differentiated thyroid cancer | differentiated thyroid cancer; insulin resistance; igfbp-3 gene polymorphisms	insulin resistance (18.2, 1.0E-4); risk of recurrence (9, 0.005); irs-1 (9, 0.005); igfbp-3 gene polymorphisms (9, 0.005); argentina (9, 0.005)
7	128	0.976	2004	oral squamous cell carcinoma; cellular inhibitor; apoptosis protein; oral squamous cell carcinoma cell lines; drug resistance | oral squamous cell carcinoma cell lines; drug resistance; cellular inhibitor; apoptosis protein; oral squamous cell carcinoma	oral squamous cell carcinoma cell lines (9.2, 0.005); cellular inhibitor of apoptosis protein 2 (9.2, 0.005); microarray (9.2, 0.005); mtt assay (9.2, 0.005); cddp (6.45, 0.05)
8	126	1	2007	larynx cancer; dna methylation; plant polyphenols; akt pathway; hep-2 cells | akt pathway; hep-2 cells; laryngeal cancer; larynx cancer; dna methylation	erk signaling (8.82, 0.005); akt pathway (8.82, 0.005); inpp4b (8.82, 0.005); chemoprevention (8.82, 0.005); hep-2 cells (8.82, 0.005)

Taken together, the co-citation and clustering analysis of the literature in the field of head and neck cancer drug resistance research provided us with a deep insight into the research hotspots and the evolution of the key #0 to #8 clusters.

### 3.6 Analysis of journals and co-cited journals

**FIGURE 8:**
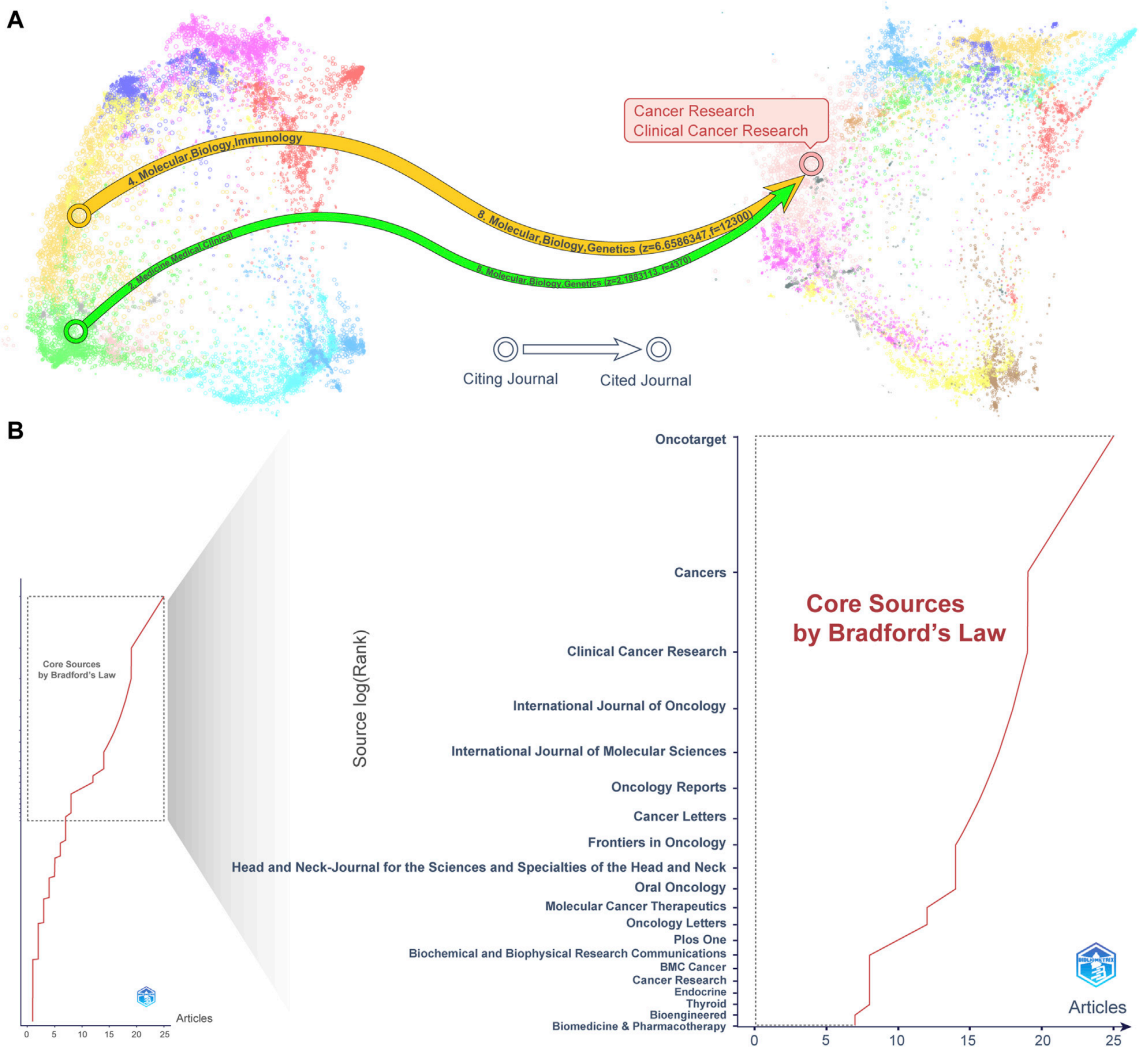
Journal analysis of influential publications. **(A)** A dual-map overlay shows journals. Cancer Research and Clinical Cancer Research were the leading journals cited. **(B)** By applying Bradford’s Law to the analysis, we identified the top 20 influential journals.

We analyzed journals in the field of drug resistance in head and neck cancer through Citespace’s “Overlay Maps”. Overlap analysis helps identify high-impact journals and papers to provide research direction. Figure 8A provides the necessary knowledge base for citing journals. The yellow #4 and green #2 paths in the journal biplot overlay mapping indicate that research published in journals related to “Molecular, Biological and Immunological” and “Medical, Medical and Clinical”, respectively, is usually cited in journals in the “Molecular, Biological and Genetic” field, which is colored #8 in pink. Specifically, the cited articles in “Molecular, Biology and Genetics” were primarily from Cancer Research and Clinical Cancer Research journals. In addition, we counted the impact factors, JCR quartiles, and open access (OA) information of the top 20 cited journals in Supplementary Table S8. These findings highlight the interconnectedness of different fields in the study of drug resistance in head and neck cancer. They also highlight the importance of interdisciplinary research in advancing scientific knowledge.

Furthermore, we analyzed the highly published and influential journals in the field of drug resistance in head and neck cancer. We used the “Bradford’s Law” module of the bibloshiny tool. According to Bradford’s Law, a small number of core journals contribute the majority of research output in the field. In contrast, most journals contribute relatively little. The left side of Figure 8B presents the overall picture and the right side shows the core journals. There are 20 core journals on the panel, of which the TOP3 journals are Oncotarget, Cancers, and Clinical Cancer Research. Further, we count the publication volume, H-index, impact factor, partitioning, and OA index of these 20 core journals in the field in Table 4. The first thing to note is that the journal Oncotarget has open access (OA) attributes and ranks first in the field with 25 articles and 19 H-indexes in the area of head and neck cancer drug resistance. However, it is worth pointing out that the journal is not included in the Science Citation Index (SCI) journal category. Second, Clinical Cancer Research demonstrated the highest overall drug resistance in head and neck cancer. This was in terms of number of articles, H-index, impact factor, and partitioning. Finally, of these 20 core journals, 11 are in the high impact factor quartile (Q1), 6 are in the medium quartile (Q2), and 2 are in the lower quartile (Q3). Of these, eight journals have an impact factor of more than 5, while seven are open access (OA) journals. Taken together, we gained more comprehensive access to the latest research findings about drug resistance in head and neck cancer. We did this by focusing exclusively on core journals in this area.

**TABLE 4:** Top 20 journals: H-index, JCR impact factor, quartiles and OA details.

Rank	Journal	Counts	H-index	If (JCR 2022)	JCR quatile	OA
1	ONCOTARGET	25	19	-	-	Yes
2	CANCERS	19	8	5.2	Q1	Yes
3	CLINICAL CANCER RESEARCH	19	17	11.5	Q1	No
4	INTERNATIONAL JOURNAL OF ONCOLOGY	18	14	5.2	Q1	No
5	INTERNATIONAL JOURNAL OF MOLECULAR SCIENCES	17	8	5.6	Q1	Yes
6	ONCOLOGY REPORTS	16	12	4.2	Q2	No
7	CANCER LETTERS	15	13	9.7	Q1	No
8	FRONTIERS IN ONCOLOGY	14	7	4.7	Q2	Yes
9	HEAD AND NECK-JOURNAL FOR THE SCIENCES AND SPECIALTIES OF THE HEAD AND NECK	14	10	2.9	Q1	No
10	ORAL ONCOLOGY	14	11	4.8	Q1	No
11	MOLECULAR CANCER THERAPEUTICS	12	11	5.7	Q2	No
12	ONCOLOGY LETTERS	12	8	2.9	Q3	No
13	PLOS ONE	10	8	3.7	Q2	Yes
14	BIOCHEMICAL AND BIOPHYSICAL RESEARCH COMMUNICATIONS	8	5	3.1	Q2	No
15	BMC CANCER	8	6	3.8	Q2	Yes
16	CANCER RESEARCH	8	8	11.2	Q1	No
17	ENDOCRINE	8	5	3.7	Q3	No
18	THYROID	8	7	6.6	Q1	No
19	BIOENGINEERED	7	5	4.9	Q1	Yes
20	BIOMEDICINE and PHARMACOTHERAPY	7	6	7.5	Q1	No

## 4 Discussion

### 4.1 Pharmacotherapeutic strategies for head and neck cancer: Present and future

**FIGURE 9:**
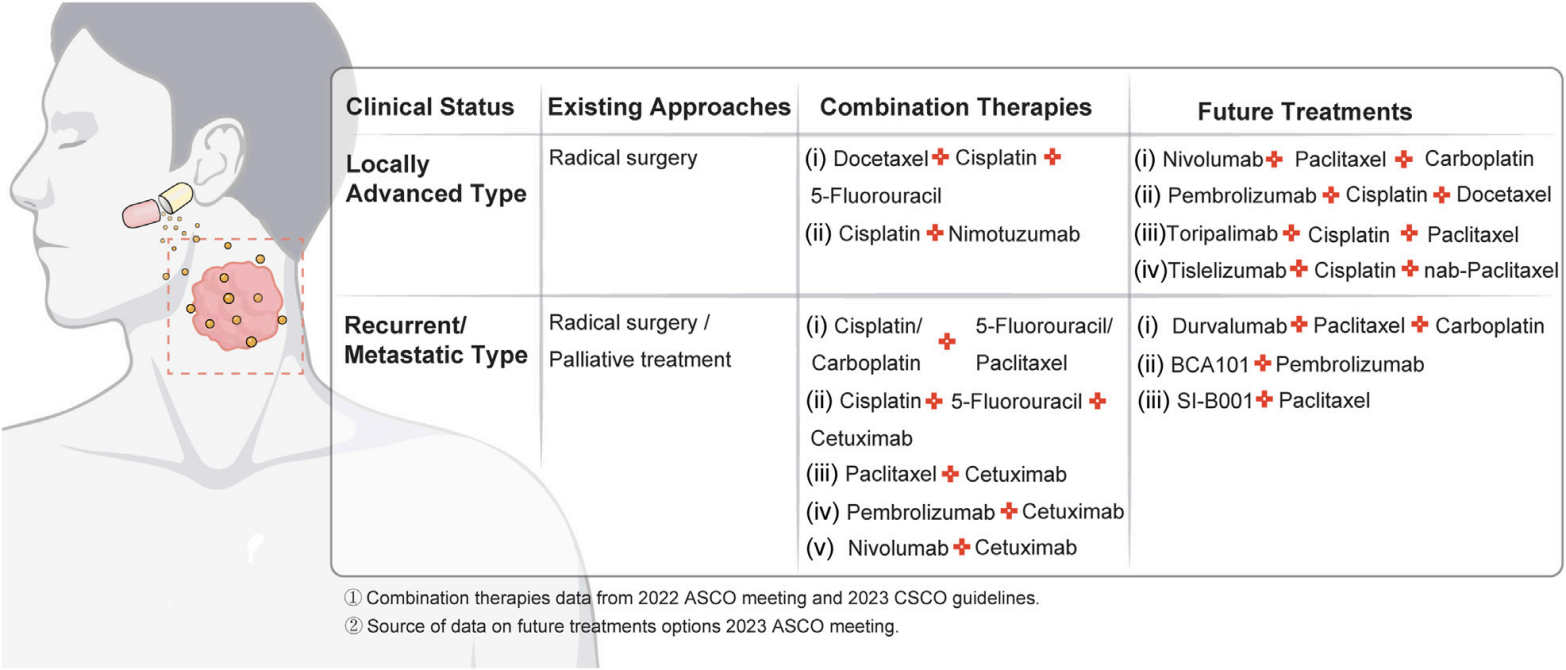
Perspectives on current and future drug therapies for head and neck cancer.

For patients with locally advanced and recurrent metastatic head and neck cancer, surgery and drug combination therapy have become the current mainstream recommended regimen. In Figure 9, we have combined the 2022 American Society of Clinical Oncology (ASCO) meeting and the 2023 Chinese Society of Clinical Oncology (CSCO) guidelines on head and neck cancer to provide an overview of current treatment strategies and drug combination options. In addition, we refer to new advances in head and neck cancer treatment presented at the ASCO 2023 meeting to guide future treatment regimens.

In the area of drug therapy for localized head and neck cancer, four academics’ protocols were included in the category of “future therapies”. Prof. Rosenberg says a neoadjuvant regimen based on nivolumab in combination with paclitaxel and carboplatin has shown significant survival and local control benefits in patients with highly advanced HPV-negative squamous head and neck cancer (Rosenberg et al., 2023). Meanwhile, the experimental II regimen introduced by Prof. Ferrarotto in the field of laryngeal cancer treatment showed excellent advantages in terms of laryngeal preservation rate, recurrence rate and survival rate (Ferrarotto et al., 2023). In addition, experimental regimens iii and iv proposed by Chinese doctors Xiao Min and Gui Lin have shown significant efficacy in the treatment of localized head and neck cancer (Gui et al., 2023; Ou et al., 2023).

The protocols of three authors have been included in the category of “future treatments” for recurrent metastatic head and neck cancers. First, a study from France treated 64 patients who could not receive cisplatin-based chemotherapy with regimen i, showing safety and efficacy in a vulnerable population (Fayette et al., 2023). Second, Prof. Hanna presented regimen ii (containing the EGFR/TGFβ bifunctional inhibitor BCA101 in combination with pembrolizumab), which showed significant efficacy in HPV-negative patients (Hanna et al., 2023). Finally, Dr. Xue Liqiong shared regimen iii (including the EGFR/HER3 bispecific antibody SI-B001 in combination with paclitaxel), which presented significant efficacy in patients who had not received paclitaxel (Xue et al., 2023). Of particular note is the growing importance of “de-chemotherapy” in the treatment of recurrent/metastatic head and neck cancer. In the 2023 edition of the CSCO Head and Neck Cancer Treatment Guidelines, targeted therapy in combination with immunotherapy is recommended as a level III therapy. This indicates that this treatment modality is being explored. As a result, recurrent/metastatic head and neck cancer may move toward a " De-chemotherapy " era in the future. These options are included in the category of “future treatments” for locally advanced head and neck cancer.

Additionally, targeted therapy combined with immunotherapy is recommended as a level III recommendation in the CSCO Head and Neck Cancer Treatment Guidelines 2023. This indicates that this treatment modality is being explored. As a result, head and neck cancer treatment may progress toward a " De-chemotherapy " era in the future.

### 4.2 Global growth trends and drug resistance research in head and neck cancer

We statistically analyzed head and neck cancer drug resistance trends from 2000 to 2023. We paid particular attention to the significant growth in recent years. Annual citation trends show three phases of fluctuation, plateauing, and decreasing over time. These phases may be due to low-quality research, fragmentation of the field, citation lag, changes in research direction, and potential data deficiencies or short-term fluctuations.

Continued global investment and in-depth exploration of basic drug resistance research in head and neck cancer has provided crucial impetus for advancing drug resistance research in head and neck cancer. In recent years, worldwide research on drug resistance in head and neck cancer has made progress, focusing on the following three aspects.

(1) Targeted therapy: This therapy kills tumor cells by inhibiting specific targets of tumor cells. Although targeted agents such as erlotinib and cetuximab have gained approval in the treatment of head and neck cancer, patients are often challenged by resistance to the targeted agents (Ratushny et al., 2009). Studies have revealed resistance mechanisms, including genetic mutations and signaling pathway reorganization (Chen et al., 2021; Li et al., 2022; Chan et al., 2023). These findings highlight limitations to therapy effectiveness. More in-depth studies are urgently needed to find effective strategies to overcome drug resistance to improve the survival of head and neck cancer patients.
(2) Immunotherapy: Currently, the main treatments are PD-1/PD-L1 inhibitors (Miraki Feriz et al., 2023), CTLA-4 inhibitors (Tasaki et al., 2023), NK cell therapy (Ayuso et al., 2023; Jung et al., 2023), and a combination of these approaches (Sperling et al., 2023). In addition, vaccine therapies aim to stimulate the patient’s defense system to respond specifically to the tumor (Chaudhary et al., 2023b; Santegoets et al., 2023). Research into head and neck cancer vaccines is ongoing. However, immune selection eliminates T-cell-sensitive subclones and drug-resistant subclones. It has been found that this may be caused by multiple immune escape mechanisms that limit therapeutic efficacy (Lee and Allen, 2020). To overcome immune resistance, researchers need to gain a deeper understanding of molecular mechanisms and develop more targeted therapeutic strategies. This will improve immunotherapy durability and efficacy for head and neck cancer.
(3) Novel chemotherapeutic drugs: In recent years, novel chemotherapeutic drugs have been developed, such as salvia divinorum extract (Yang et al., 2017), tetrandrine (Chang et al., 2023), fluorouracil derivatives (Vermorken et al., 2011; Oertel et al., 2012) and anti-angiogenic drugs. Research shows that these novel chemotherapeutic drugs can effectively improve head and neck cancer patients’ therapeutic effect (Murányi et al., 2023).

Furthermore, individualized treatment, including stem cell (Küçükgüven and Çelebi-Saltik, 2021; Byun et al., 2022) and gene therapy. Medical professionals can use systematic genomic analysis to understand cancer characteristics and develop personalized treatment plans. In summary, global research on drug resistance in head and neck cancer has progressed, yet significant challenges remain. Future research should focus on innovating more effective treatments to improve patient prognosis.

### 4.3 Contributions by states, institutions and authors

This paper statistically analyzes international collaborations in head and neck cancer drug resistance research at three levels: country, institution and author. Firstly, China, the United States and Japan lead in head and neck cancer drug resistance research. This is with the number of publications and citation frequency ranking among the top three. Among them, China has an absolute advantage in the number of publications, and the United States has an obvious advantage in the frequency of citations. Secondly, in terms of institutions, China, the United States, and Japan are the main contributors to drug resistance research in head and neck cancer. China Medical University is the leading university in China. The United States plays a key role in international collaborations. M.D. Anderson Cancer Research Center is very significant in the field because it provides key support between the various clusters. Finally, in terms of authors, the Top10 authors, Grandis Jennifer R and Yang Jai-Sing lead the volume of publications, while Osmak Maja, Kim Eun Hye, Roh Jong-Lyel, and Lu Chi-Cheng are noted for their excellent citation performance. Through LLR clustering analysis, the TOP10 authors focused on five major research directions. This demonstrated their profound impact on head and neck cancer drug resistance research. Detailed statistics on the TOP10 authors’ publication volume, academic index, and influence in the field highlight their notable academic and clinical contributions.

### 4.4 Research hotspots identified through keyword and literature clustering

Head and neck cancer drug resistance studies revealed research hotspots through keyword co-occurrence analysis. First, VOSViewer counted and visualized keywords. This indicated that treatment method, drug resistance type, and research topic were the three major categories of keywords. Second, LLR and LSI clustering analysis and visualization were used to obtain 16 clusters. These clusters included multidrug resistance in nasopharyngeal carcinoma, the application of RT-PCR in drug resistance research, and the relationship between coenzyme Q-related genes and drug resistance. Finally, the annual keyword frequency heatmap revealed therapeutic focus (Paclitaxel, Immunotherapy, Cisplatin Resistance, Cetuximab), emerging themes (MicroRNA, Metastasis, Ferroptosis, AKT), and motor themes (Cetuximab Resistance, Cisplatin-resistance, Erlotinib, Cell Hypoxia), providing a comprehensive understanding of hotspots and trends in head and neck cancer resistance research. Specifically, emerging areas of cetuximab resistance research in head and neck cancer involve EFNB2 (Chaudhary et al., 2023a), NRG1 (Iida et al., 2022), TRIP13-pY56 (Banerjee et al., 2022), and mathematical resistance prediction models (Cárdenas et al., 2022). Furthermore, resveratrol (Chang et al., 2021), miR-196a (Qin et al., 2019), ferroptosis (Roh et al., 2017; Shin et al., 2018), and photodynamic therapy (Lu et al., 2014) have been investigated specifically as agents for reversing cisplatin resistance in head and neck cancer.

Literature co-citation cluster analysis deepened drug resistance research knowledge structure and focused on head and neck cancer. First, citespace clustered literature keywords and titles to generate nine clusters. It labeled the cluster labels using LSI and LLR algorithms. Second, the hotspots and field evolution of drug resistance research in head and neck cancer were revealed by analyzing the taxon labels and their dependencies. #1 focuses on cancer stem cells, #2-#6 reflect hot topics, while #7 involves Ferroptosis and Cisplatin Resistance, and #8 may involve laryngeal squamous carcinoma drug resistance with the ERK pathway (Kim et al., 2012). Chemotherapeutic agents may increase stem-like cells in cancer tissue (Dorna and Paluszczak, 2023). Therefore, combining conventional chemotherapeutic agents with stemness modulators inhibits cancer cell proliferation and reduces recurrence. By combining a sphingosine kinase one antagonist (FTY720) with paclitaxel, the levels of stemness-associated proteins in oral squamous carcinoma cells can be effectively reduced, which in turn achieves anti-cancer effects (Torres et al., 2024). Finally, the Citespace “Cluster Dependencies” module was used to visualize the dependencies between clusters, simplifying the evolutionary pattern and revealing the research focus trajectory (Li and Li, 2024). This provides a deep insight into the hot trends and evolutionary relationships between taxa in head and neck cancer drug resistance research.

### 4.5 Head and neck Cancer Research: The most influential journal

We analyzed journals in the field of drug resistance in head and neck cancer through Citespace’s “Overlay Maps”, revealing the importance of interdisciplinary research. The overlap analysis between cited journals showed that research and citations published in the fields of “Molecular, Biology and Immunology” and “Medicine, Medical and Clinical” were mainly concentrated in journals related to “Molecular, Biology and Genetics”, emphasizing the interconnections between different fields. In addition, we analyzed the core journals using Bradford’s Law and found that Oncotarget, Cancers, and Clinical Cancer Research were the main influential journals in the field of drug resistance in head and neck cancer, with Clinical Cancer Research showing the highest level of overall strength, and Oncotarget demonstrating the highest level of open access strength, and Clinical Cancer Research showing the highest level of open access strength. Oncotarget was also noted for its open access attributes and high impact. The specific attributes of these core journals are critical for obtaining the latest research results on drug resistance in head and neck cancer.

### 4.6 Research strengths and limitations

This study has the following strengths.

(1) To the extent of our knowledge, this study is the first bibliometric literature that comprehensively assesses global drug resistance studies in head and neck cancer.
(2) We used the most reliable WOSCC database for bibliometric analysis (Liu et al., 2024b; Zhang et al., 2024). This database has broad coverage, high-quality literature, standardized data, and comprehensive search functionality.
(3) We comprehensively used a variety of analytical tools such as Citespace, R package bibliometric, VOSviewer, SCImago Graphica, and MapEquation to econometrically analyze the literature on drug resistance in head and neck cancer, which provided comprehensive and in-depth scholarly insights to help researchers understand the research dynamics, partnerships, and hotspot trends in drug resistance in head and neck cancer in a morin-depthve way.

Shortcomings of this study.

(1) Although a single database (WOSCC) was used in this study for bibliometric analysis of head and neck cancer drug resistance studies. However, several studies have shown that this database is considered an excellent resource for bibliometric studies in various disciplines.
(2) Given the time lag in the inclusion of papers in the WOSCC database, this study could not cover all papers published in 2023. Therefore, it is impossible to present the latest research results on drug resistance in head and neck cancer.

## 5 Conclusion

This study can help researchers understand the global focus and trends in head and neck cancer drug resistance research from 2000 to December 2023. First, the statistical analysis of the 2000–2023 trends in the field of head and neck cancer drug resistance research shows that the trend of publications has increased significantly. However, the citation trend goes through three stages of fluctuation, stabilization, and decreasing year by year. Then, China, the United States, and Japan lead the head and neck cancer drug resistance research, and China Medical University has a leading position at the institutional level, and the Top 10 authors have significant outputs in the five research directions, which highlights their significant contributions in both academic and clinical aspects. Second, the keyword co-occurrence and co-citation analysis of Head and Neck Cancer Drug Resistance Research comprehensively reveals the research hotspots, trends and knowledge structure, and provides a deep insight into the evolution of the field. Finally, the “Overlay Maps” and Bradford’s Law analyses reveal the cross-impact and core journals in the field of drug resistance in head and neck cancer, providing directions for comprehensive access to the latest research results. In conclusion, this literature provides researchers with an in-depth understanding of the current status and hot trends of drug resistance research in head and neck cancer worldwide.

## Data Availability

The original contributions presented in the study are included in the article/Supplementary Material, further inquiries can be directed to the corresponding authors.
