# Supplementary material for: Decoding the research landscape of drug resistance and therapeutic approaches in head and neck cancer: a bibliometric analysis from 2000 to 2023
Source: Front Pharmacol. 2024 Apr 5;15:1375110. doi: 10.3389/fphar.2024.1375110 (PMC11026562; doi:10.3389/fphar.2024.1375110)
Supplement: Supplementary file 1 [file DataSheet1.PDF]

Supplementary Materials

Figure S1. TOP4 burst strength author. 2
Figure S2. TOP N=40 keyword cluster analysis. 3
Figure S3. TOP N=50 keyword cluster analysis. 4
Figure S4. Keyword fuzzy set theme analysis. 5
Figure S5. Title literature clustering and cluster dependency analysis 6
Table S1. National publications and standardized citations 7
Table S2. TOP10 Author Publications 8
Table S3. TOP10 Authors H-index 9
Table S4. TOP10 Authors G-index 10
Table S5. TOP10 Authors M-index 11
Table S6. Rank 11-20 number of word frequencies for treatments 12
Table S7. Rank 11-23 Number of word frequencies for themes 13
Table S8. Top20 cited journals and impact factor of JCR. 14

Figure S1. TOP4 burst strength author.

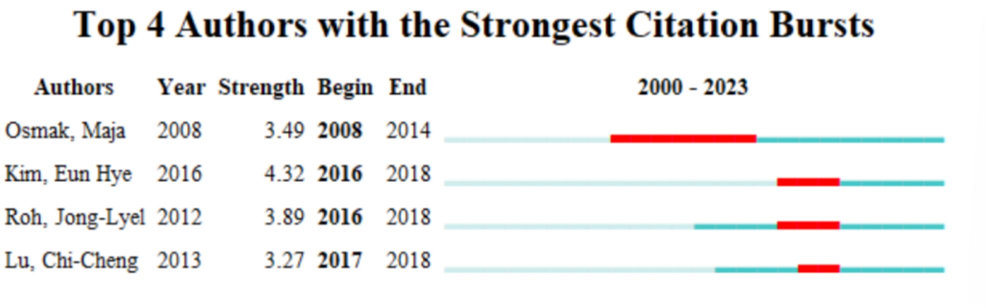

Top 4 Authors with the Strongest Citation Bursts

Authors	Year	Strength	Begin	End	2000 - 2023
Osmak, Maja	2008	3.49	**2008**	2014	
Kim, Eun Hye	2016	4.32	**2016**	2018	
Roh, Jong-Lyel	2012	3.89	**2016**	2018	
Lu, Chi-Cheng	2013	3.27	**2017**	2018	

frontiers

Figure S2. TOP N=40 keyword cluster analysis.

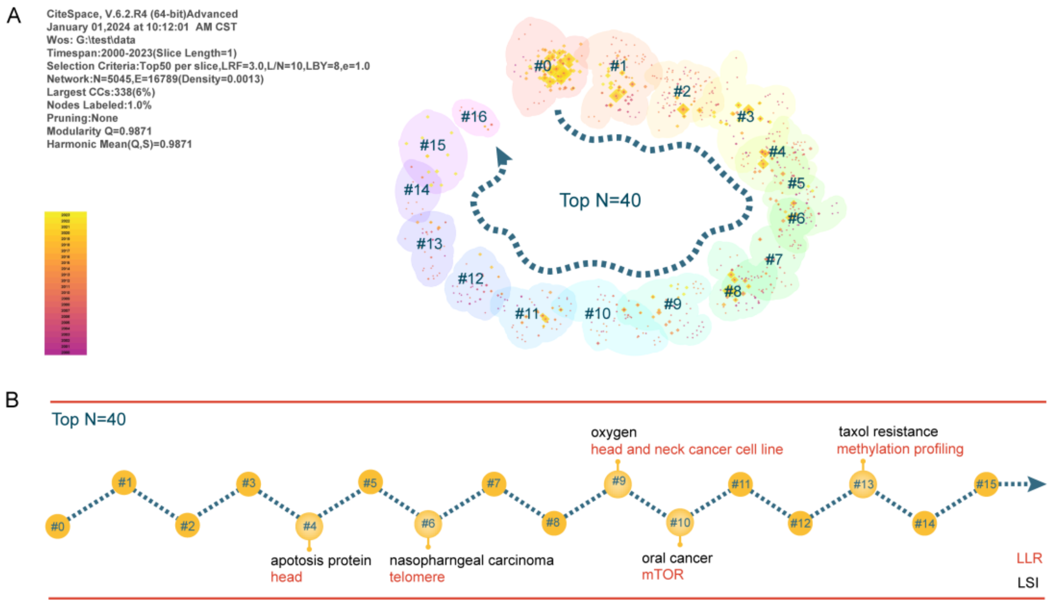

Figure S3. TOP N=50 keyword cluster analysis.

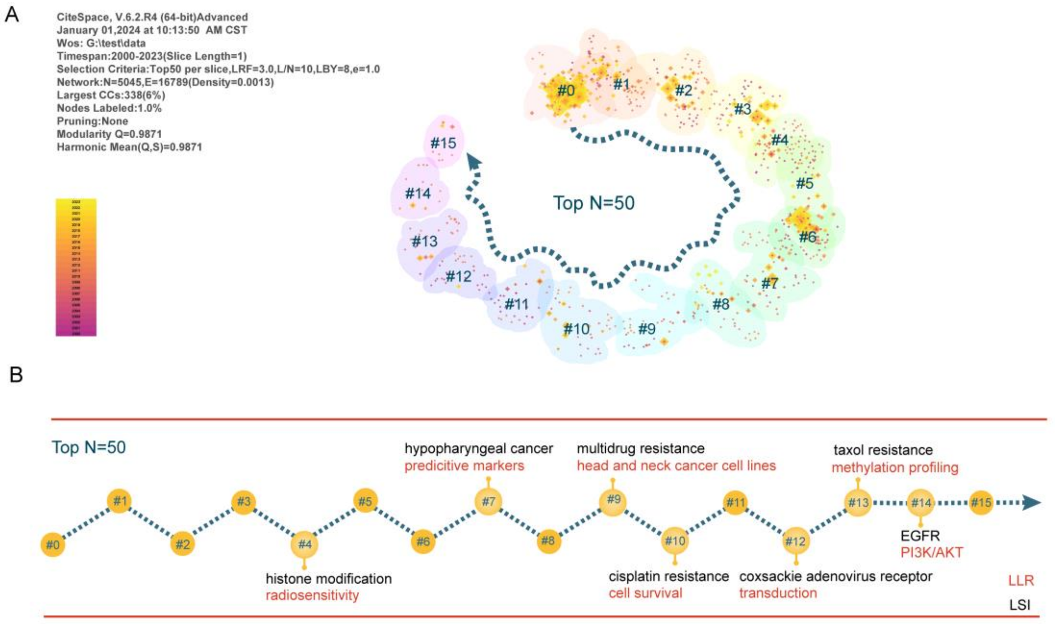

Figure S4. Keyword fuzzy set theme analysis.

The keywords were divided into 4 quadrants, where the horizontal coordinate indicates centrality and the vertical coordinate indicates density. The upper right quadrant covers significant keywords related to the Motor themes.

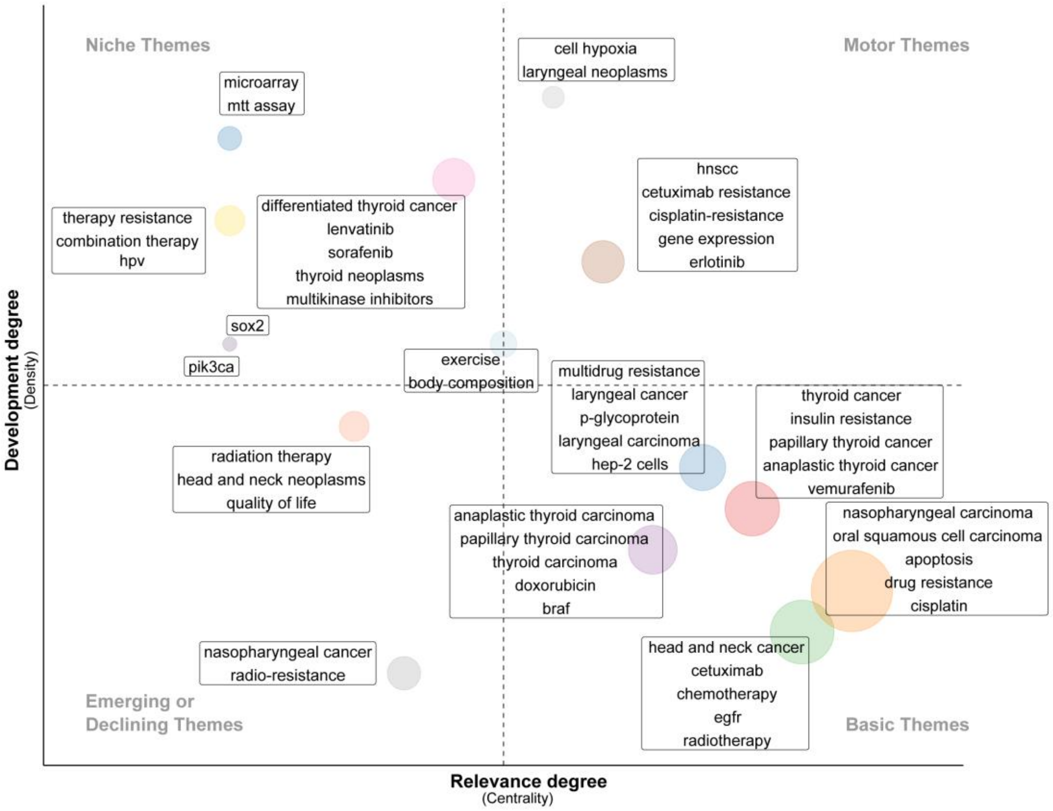

Figure S5. Title literature clustering and cluster dependency analysis

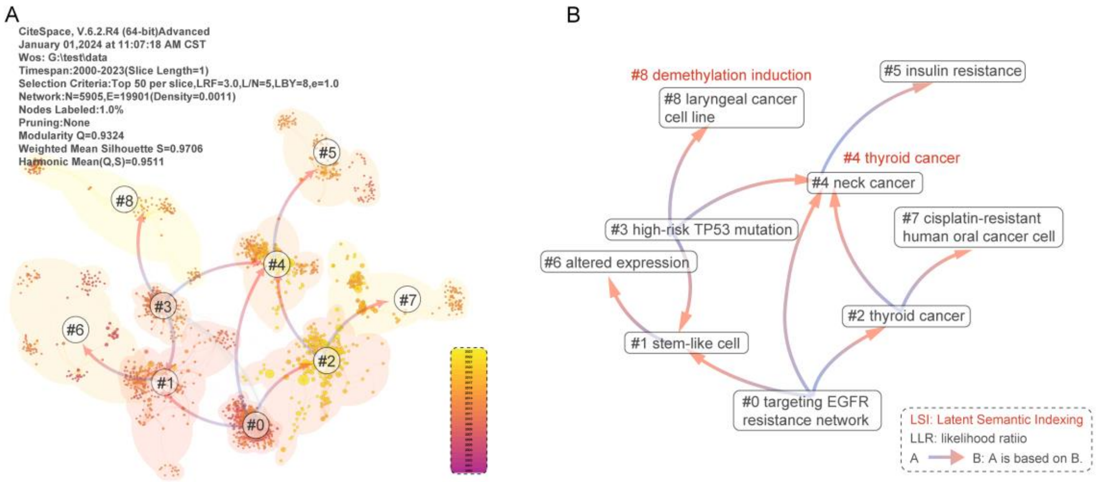

Table S1. National publications and standardized citations

Country	Counts	Normal Citation
China	386	354.908
USA	164	213.9498
Japan	63	43.7272
South Korea	45	55.7912
Italy	32	25.9362
Germany	31	29.1531
India	29	29.9469
England	16	18.2241
Spain	14	13.2652
Australia	11	15.7803
Canada	11	10.7729
France	11	12.0417
Croatia	9	3.8619
Greece	9	9.5954
Malaysia	7	4.9874
Turkey	7	6.1452
Brazil	6	9.2725
Egypt	6	32.9467
Thailand	6	3.0167
Singapore	5	5.0444
Sweden	5	4.8774

Table S2. TOP10 Author Publications

**Rank**	**Authors**	**Counts**
1	Yang Jai-Sing	13
2	Grandis Jennifer R	13
3	Li Wei	12
4	Roh Jong-Lyel	11
5	Lu Chi-Cheng	10
6	Tan Guolin	10
7	Kim Eun Hye	9
8	Osmak Maja	9
9	Shin Daiha	8
10	Nakayama Hideki	7
11	Yoshida Ryoji	7
12	Shinohara Masanori	7

Table S3. TOP10 Authors H-index

Rank	Authors	H-index
1	Yang Jai-Sing	12
2	Grandis Jennifer R	10
3	Lu Chi-Cheng	10
4	Roh Jong-Lyel	10
5	Kim Eun Hye	9
6	Li Wei	8
7	Osmak Maja	8
8	Shin Daiha	8
9	Nakayama Hideki	7
10	Yoshida Ryoji	7

Table S4. TOP10 Authors G-index

Rank	Authors	G_index
1	Yang Jai-Sing	13
2	Grandis Jennifer R	13
3	Li Wei	12
4	Roh Jong-Lyel	11
5	Lu Chi-Cheng	10
6	Tan Guolin	10
7	Kim Eun Hye	9
8	Osmak Maja	9
9	Shin Daiha	8
10	Nakayama Hideki	7
11	Yoshida Ryoji	7
12	Shinohara M	7

Table S5. TOP10 Authors M-index

Rank	Authors	M_index
1	Kim Eun Hye	1.125
2	Yang Jai-Sing	1.091
3	Shin Daiha	1
4	Lu Chi-Cheng	0.909
5	Roh Jong-Lyel	0.833
6	Li Wei	0.571
7	Grandis Jennifer R	0.556
8	Nakayama Hideki	0.538
9	Yoshida Ryoji	0.538
10	Shinohara M	0.462

Table S6. Rank 11-20 number of word frequencies for treatments

Rank	Treatment Approaches	counts
11	docetaxel	3
12	erlotinib	3
13	nivolumab	3
14	vincristine	3
15	dasatinib	2
16	palbociclib	2
17	retinoic acid	2
18	tetrandrine	2
19	temsirolimus	2
20	triptolide	2
21	valproic acid	2

Table S7. Rank 11-23 Number of word frequencies for themes

**Rank**	**Study Themes**	**counts**
11	invasion	8
12	ferroptosis	8
13	tumor microenvironment	7
14	extracellular vesicles	7
15	DNA methylation	7
16	pi3k	6
17	stat3	5
18	ras	5
19	PTEN	5
20	hypoxia	5
21	exosome	5
22	braf	5
23	bcl-2	5

Table S8. Top20 cited journals and impact factor of JCR.

Rank	Journal	Cited Counts	IF (JCR2021)	JCR quatile	OA
1	CANCER RESEARCH	1408	11.2	Q1	No
2	CLINICAL CANCER RESEARCH	996	11.5	Q1	No
3	ONCOGENE	794	8	Q1	No
4	JOURNAL OF CLINICAL ONCOLOGY	652	45.3	Q1	No
5	ONCOTARGET	627	-	-	Yes
6	PLOS ONE	554	3.7	Q3	Yes
7	JOURNAL OF BIOLOGICAL CHEMISTRY	550	4.8	Q2	No
8	NATURE	544	64.8	Q1	No
9	CELL	526	64.5	Q1	No
10	PROCEEDINGS OF THE NATIONAL ACADEMY OF SCIENCES OF THE UNITED STATES OF AMERICA	510	11.1	Q1	No
11	INTERNATIONAL JOURNAL OF CANCER	462	6.4	Q1	No
12	NEW ENGLAND JOURNAL OF MEDICINE	458	158.5	Q1	No
13	ORAL ONCOLOGY	433	4.8	Q1	No
14	CANCER LETTERS	423	9.7	Q1	No
15	JOURNAL OF CLINICAL ENDOCRINOLOGY & METABOLISM	415	5.8	Q1	No
16	NATURE REVIEWS CANCER	393	78.5	Q1	No
17	THYROID	385	6.6	Q1	No
18	BRITISH JOURNAL OF CANCER	356	8.8	Q1	No
19	ONCOLOGY REPORTS	318	4.2	Q2	No
20	INTERNATIONAL JOURNAL OF ONCOLOGY	306	5.2	Q2	No
